# Risk of incident stroke and heart disease subtypes in a nationwide cohort of Korean radiation workers

**DOI:** 10.5271/sjweh.4251

**Published:** 2025-11-01

**Authors:** Eun Shil Cha, Kyunghee Chae, Haesu Jeong, Dalnim Lee, Soojin Park, Ga Bin Lee, Minsu Cho, Hyeonseok Nam, Songwon Seo

**Affiliations:** 1 National Radiation Emergency Medical Center, Korea Institute of Radiological and Medical Sciences, Seoul, Republic of Korea.; 2 Korea Cancer Center Hospital, Korea Institute of Radiological and Medical Sciences, Seoul, Republic of Korea.

**Keywords:** circulatory disease, Korea, low-dose radiation, occupational exposure

## Abstract

**Objectives:**

Previous studies have explored the association between low-dose radiation and circulatory diseases, but few have focused on stroke and heart disease subtypes. This study aimed to investigate subtype-specific risks among Korean radiation workers.

**Methods:**

We analyzed data from 186 233 workers enrolled in the Korean Radiation Workers Study cohort. Individual radiation exposure was assessed using personal dose data, and organ-specific doses were reconstructed. Using the National Health Insurance Database, circulatory diseases were identified based on validated operational definitions developed for six subtypes. Sex- and age-standardized incidence ratios (SIR) were estimated. Dose–response relationships were assessed by estimating the relative rates (RR) and excess relative risks (ERR) per 10 mGy, adjusting for the sex, attained age, birth year and calendar year. Additional confounders in the ERR models included income level, smoking, blood pressure, blood glucose level, and body mass index.

**Results:**

Ischemic heart disease has the highest incidence (367.6 per 100 000 person-years), followed by heart failure and ischemic stroke. The overall incidence rates of most subtypes were lower among radiation workers than the general population, reflecting a healthy worker effect (SIR range: 0.70–0.90). No statistically significant positive dose–response relationships were observed for all six subtypes; ERR estimates were generally negative, with the highest point estimate observed for hemorrhagic stroke (ERR per 10 mGy: 0.014; 95% confidence interval -0.049–0.077).

**Conclusions:**

While no clear radiation-related risk was observed, the findings highlight the importance of subtype-specific analysis and the need for refined outcome definitions and longer follow-up periods to clarify potential low-dose radiation effects.

Stroke and heart disease are major contributors to global disease burden. In 2021, ischemic heart disease (IHD) and stroke were the second and fourth leading causes of disability-adjusted life years (DALY) worldwide, accounting for 188.3 million and 160.4 million DALY, respectively (1). In East Asia, including Korea, age-standardized stroke incidence has stagnated or even increased since 2015, contrary to the declining trends observed in other regions (2). Recent evidence suggests an increasing trend in stroke incidence among young adults aged <55 years. This trend is thought to be driven by a growing prevalence of modifiable risk factors, such as high body mass index (BMI), elevated blood pressure (BP), high fasting glucose, and environmental exposure (2). Circulatory diseases are the second leading cause of death in South Korea. In 2023, 18 127 and 14 028 deaths from cerebrovascular diseases (CeVD) and IHD occurred, accounting for approximately 5.1% and 4.0% of all deaths, respectively (3). Moreover, in 2022, the incidence rates of stroke and myocardial infarction based on hospitalization episodes in Korea were reported to be 171.7 and 61.7 per 100 000, respectively (4).

Radiation-induced damage to the circulatory system from high doses (eg, from radiotherapy) has long been recognized (5, 6). Such exposure can affect multiple cardiac structures, including the pericardium, myocardium, valves, and coronary vessels, and lead to both macrovascular and microvascular injuries through endothelial cell depletion and fibrosis (5). Inflammation, oxidative stress, and endothelial dysfunction have been implicated as underlying mechanisms in radiation-associated circulatory diseases (6). However, evidence from the Life Span Study of Japanese atomic bomb survivors indicated elevated mortality risks from circulatory diseases, even at doses <1–2 Gy (7), and epidemiological studies have increasingly shown risks associated with low occupational or environmental exposures. While these studies have provided valuable insights, owing to inconsistencies across studies and unclear biological mechanisms at low doses, evidence is insufficient to establish a causal relationship at doses <1–2 Gy (8).

More recently, however, meta-analyses have revealed significantly elevated risks of circulatory diseases, including IHD, among occupationally exposed radiation workers, even at low-to-moderate doses (9). Consistent with these findings, the International Nuclear Workers Study (INWORKS) has reported increased risks of circulatory disease mortality among radiation workers exposed to protracted low-dose radiation (10). Despite these emerging findings, interpretation of the risks at low doses requires caution. These associations may be influenced by potential confounding factors, disease misclassification, or exposure measurement errors (8, 11, 12). A systematic evaluation of outcome assessment errors, such as loss to follow-up, under- or over-ascertainment of disease cases, and misclassification, suggested that these errors may have biased risk estimates toward the null, whereas differential errors potentially affecting the results were identified in a few others (13). In addition, although combining disease subtypes may enhance the statistical power in risk evaluations, broader disease categories (such as overall heart disease) should be interpreted with caution because of the heterogeneity in radiation-induced pathogenesis across subtypes (12).

To better elucidate the relationship between occupational radiation exposure and circulatory disease, recent literature has emphasized the importance of incorporating comprehensive information on clinical, lifestyle, and behavioral factors, as well as utilizing large-scale administrative health data to control for potential confounding (9). Incident-based outcome analyses are particularly useful for cardiovascular diseases, as many affected individuals survive for extended periods after disease onset. Thus, incidence data may better reflect the true burden and timing of disease following exposure. Incidence-based studies have also been recommended for examining exposure–disease latency in radiation epidemiology (9).

Therefore, we analyzed data from the Korean Radiation Workers Study (KRWS), a nationwide cohort study established by linking individual radiation dose monitoring data with large-scale administrative health data, including data from the National Health Information Database (NHID) (14). Previous studies on occupational radiation exposure and circulatory diseases have primarily focused on overall circulatory outcomes or broad categories such as CeVD and IHD, with limited analyses stratified by stroke or heart disease subtype (15, 16). These earlier Korean studies, moreover, reported little evidence of a significant positive association between occupational radiation exposure and circulatory diseases, which is somewhat contrary to the conclusions drawn in a recent review (9). To address these gaps, we aimed to investigate the association between occupational radiation exposure and the risk of developing specific subtypes of circulatory diseases. We also aimed to estimate the incidence of stroke and heart disease subtypes in the KRWS cohort using validated operational definitions, comparing these rates with those observed in the general Korean population.

## Methods

### Data sources and study population

The study population was derived from the KRWS, a nationwide cohort study of occupationally exposed workers. It includes individuals registered with the Nuclear Safety and Security Commission under the Nuclear Safety Act, whose radiation exposure has been monitored through personal dosimeters. Individual radiation dose data were obtained from the Central Registry for Radiation Workers Information, which comprises recorded quarterly measurements of personal dose equivalent [H_p_ (10)] for all radiation workers since 1984. As of 2022, the registry included data of 196 379 workers. The facility variable was defined as a mutually exclusive classification based on workplace type, with each worker assigned to the facility where they worked the longest. These classifications included: (i) nuclear power plants (NPP) involved in energy production; (ii) industrial settings representing companies engaged in economic activities such as production and sales of radioactive isotopes; (iii) educational institutions (high schools, universities, and graduate schools); (iv) non-destructive testing (NDT) facilities using radioactive isotopes for industrial testing; (v) medical institutes comprising hospitals (national, public, and university hospitals) and clinics (excluding diagnostic medical radiation workers with x-ray generator–related occupations); (vi) research institutes (national, public, or private enterprise-affiliated); (vii) public institutes fulfilling legal responsibilities; and (viii) military organizations including the army, navy, and air force.

To estimate organ-specific doses, individual doses were multiplied by two conversion coefficients provided by the International Commission on Radiological Protection (ICRP) (17). The following equation can express this organ dose reconstruction procedure:


$$D_{T}=H_{p}\left(10\right)\times [\frac{D_{T}}{K_{a}}/\frac{H_{p}(10)}{K_{a}}]$$

Where D_T_ is the organ absorbed dose (Gy), H_p_ (10) is the personal dose equivalent (Sv), and H_p_ (10)/K_a_ and D_T_/K_a_ are the dose conversion coefficients for air kerma-to-H_p_ (10) (Sv/Gy) and air kerma-to-organ dose (Gy/Gy), respectively. The conversion coefficients vary based on the energy of the radiation and the body’s orientation in relation to the direction of the radiation, which was determined by exposure scenarios for different occupational groups. The methodology for organ dose reconstruction for the KRWS is described in detail elsewhere (18). We used heart doses in the analysis for cardiovascular diseases such as IHD and acute myocardial infarction (AMI), and brain doses for cerebrovascular diseases such as ischemic stroke (IS) and hemorrhagic stroke (HS).

The cohort data were linked to the NHID from 2002 to 2021, provided by the National Health Insurance Service (NHIS), is a population-based data repository that covers over 50 million individuals in Korea. The NHID consists of sub-databases on eligibility, health screening, healthcare utilization, long-term care insurance, and healthcare provider (19). The eligibility database contains information on sociodemographic characteristics, disability, and date of death. The health screening database comprises detailed lifestyle questionnaires, laboratory results, and anthropometric measurements, with a participation rate of 74.8% in 2014 (19). The healthcare utilization database contains comprehensive information on diagnoses coded based on the International Classification of Diseases, Tenth Revision (ICD-10), inpatient and outpatient visits, prescriptions, and procedure codes used for insurance reimbursement. Each insured individual receives a unique resident registration number to track their health insurance claims. These databases are internally linked using de-identified join keys, enabling comprehensive and longitudinal health information for the entire Korean population.

Workers with any circulatory disease diagnosis prior to dose monitoring or in 2002 were excluded to eliminate prevalent cases. Those who died before 2002 were also excluded due to lack of claims data before that year. Follow-up began on the later of either the start of dose monitoring or 1 January 2003 (applying a one-year washout period), and continued until diagnosis, death, or 31 December 2021, whichever came first. The final analytical cohort comprised 186 233 workers.

### Operational definition of stroke and heart disease subtypes

In epidemiological studies using administrative databases, operational definitions–predefined algorithms based on diagnostic codes and additional information such as clinical records, prescriptions, and procedures–are used for accurate disease identification. Although the NHID was originally established for reimbursement purposes, it has been widely used in research with validated definitions to enhance diagnostic accuracy of circulatory disease (20, 21). Since 2000, several studies have estimated the incidence of AMI and stroke using the NHID or hospital data, with incidence estimates varying depending on the definitions applied (20, 21). In 2024, the Korea Disease Control and Prevention Agency published the first official statistics on cardio-cerebrovascular diseases based on epidemiological diagnostic criteria, including detailed data on incidence and fatality rates (4).

In this study, we defined six subtypes of circulatory diseases—HS, IS, IHD, AMI, valvular heart disease (VHD), and heart failure (HF)—based on existing guidelines and previous literature. Definitions were reviewed and finalized through consultations with cardiologists to ensure clinical relevance. Table 1 summarizes the criteria used for each subtype. Procedure codes for diagnostic tests and interventions are listed in supplementary material (www.sjweh.fi/article/4251) table S1. IS (ICD-10: I63–I64) and HS (I60–I62) were identified from hospitalizations, with the codes recorded as a primary or secondary diagnosis. IHD (I20–I25) required hospitalization or ≥2 outpatient visits within a year, along with relevant procedural records. AMI was defined by code I21–I23. VHD (I05–I08, I09.1, and I34–I39) included both rheumatic and non-rheumatic valvular disorders. HF was defined by code I50.

**Table 1:** Operational definitions of subtypes of stroke and heart disease. [CT=computed tomography; MRI=magnetic resonance imaging; PCI=percutaneous coronary intervention; CABG=coronary artery bypass graft].

	ICD-10*	Type and number of diagnosis	Diagnostic test or therapy
Ischemic stroke	I63–I64	Admission ≥1	Brain imaging (CT or MRI), or cerebral angiography or percutaneous thrombectomy
Hemorrhagic stroke	I60–I62	Admission ≥1	Brain imaging (CT or MRI) or cerebral angiography
Ischemic heart disease	I20–I25	Admission ≥1 or outpatient ≥2	Coronary angiography, PCI or CABG
Acute myocardial infarction	I21–I23	Admission ≥1 or outpatient ≥2	Coronary angiography, PCI or CABG
Valvular heart disease	I05–I08, I09.1, I34–I39	-	-
Heart failure	I50	-	-

*Included in principle and additional codes.

### Non-radiation risk factors and socioeconomic status

Data on non-radiation risk factors for disease were obtained from the health screening sub-database of the NHID. The NHIS conducts general health screenings annually for manual workers and biennially for non-manual workers. The screening data include BMI (kg/m^2^), BP (mmHg), fasting blood glucose level (mg/dl), and self-reported lifestyle factors such as smoking and alcohol consumption. In this study, data from health screenings conducted between 2002 and 2021 were used. The screening record closest to the year of disease diagnosis was selected for each participant. Among participants with available screening data, 43.7% completed the health screening in the same year as their diagnosis, and 79.1% had data from within three years of diagnosis. In total, 9.3% of participants had no health screening records during the study period. As a proxy for income level, the variable of insurance contribution was obtained from the eligibility sub-database. Insurance contributions are calculated based on monthly wages and equally shared between the employees and employers. NHIS premiums were categorized into 20 vigintiles, with an additional category for medical benefits.

### Statistical analysis

To compare the incidence of circulatory disease subtypes between radiation workers and the general population, we calculated age- and sex-standardized incidence ratios (SIR) using the National Sample Cohort (NSC). For this, we first estimated age-, sex-, and calendar year-specific incidence rates for each disease subtypes based on the NSC data. These rates were then applied to the corresponding person-years of radiation workers in each stratum to calculate the expected number of cases. The SIR was computed as the ratio of the observed to the expected number of cases. The NSC, developed by the NHIS of Korea, comprises approximately 1 million individuals (2.2% of the Korean population in 2002) selected through stratified random sampling based on age, sex, insurance status, and income level, and was followed from 2002 to 2019. Its high representativeness and public accessibility make it a practical and reliable data source for nationwide epidemiologic studies. Dose–response relationships were assessed by estimating relative rates (RR) and excess relative risks (ERR) per unit dose using Poisson regression for grouped data. All ERR analyses were based on the cohort of 186 233 individuals with available data on dose, follow-up period, and relevant covariates. Case counts and person-years were stratified by dose, sex, year of birth, five-year intervals of attained age, and follow-up period. Cumulative heart or brain dose (mGy) was categorized a priori (0, ≤1.0, ≤2.5, ≤5.0, ≤10.0, ≤20.0, ≤50.0, >50.0) and modeled as a time-dependent variable. To evaluate potential confounding, further stratification was conducted for smoking (never, ex-smoker, and current smokers), BMI [<18.5=underweight; 18.5–24.9=normal; 25.0–29.9=overweight; and ≥30.0=obese (kg/m^2^)], BP [systolic/diastolic: <120/<80=normal; 120–140/80–90=pre-hypertension; and ≥140/≥90=hypertension (mm Hg)], fasting blood glucose level [<100 mg/dl=normal; 100–125=prediabetes; and ≥126=diabetes (mg/dl)], and income [low=0 (medical aid and beneficiary)–6^th^ quantile of insurance amount; medium=7^th^–13^th^ quantile; and high=14^th^–20^th^ quantile].

We employed the AMFIT module in Epicure software for parameter estimation, with significance tests and 95% confidence intervals (CI) based on likelihood profiles. The primary models used are ERR models of the form


$$\lambda_{0}\left(s,a,c,b\right)[1+ERR(d)]$$

where $\lambda_{0}$ is the baseline, or background rate (ie, the rate for people with zero dose), which depends on sex (s), attained age (a), calendar year (c), birth year (b). The function ERR(d) describes the RR change associated with dose (d). Log-likelihood tests were used to compare the two models and determine which model fit better. To account for latency between radiation exposure and disease onset, cumulative doses were lagged by 0, 5 or 10 years. These lag periods were selected based on prior studies and the follow-up time available in our cohort. For the main analyses, we selected the 10-year lagged model. Based on this model, we conducted a series of sensitivity analyses by further adjusting for employment duration and individual lifestyle-related variables. To control for potential confounding, models additionally included smoking status, income, BMI, BP, and fasting blood glucose levels. Sensitivity analyses were conducted by excluding workers who had been employed for <1 year. As the dataset did not cover the entire dose monitoring or disease follow-up period for all participants, we conducted stratified analyses using key calendar cutoffs, specifically, 1984 (the start of dose monitoring) and 2002 (the beginning of disease follow-up). To evaluate potential differences in risk by dose range, we further restricted the analyses to workers with cumulative doses below 200, 100, 50, and 20 mGy. All main and sensitivity analyses were based on linear dose-response models with a 10-year lag. To evaluate potential non-linearity in the dose–response relationship, we additionally fitted linear-quadratic excess relative risk models for each outcome. Model fits were compared using likelihood ratio tests.

## Results

### Participant characteristics

Table 2 presents the general characteristics of the total study population by circulatory disease subtype. A mean follow-up duration of 13.3 years yielded 2 483 113 person-years. The incidence of circulatory diseases varied by subtype, with IHD being the most common (8562), followed by HF (5892) and IS (1888). The majority of the population were male (83.4%), with higher proportions in disease groups (eg, 97.6% for AMI). The mean age of the total population was 45.6 years, with higher ages in disease groups (eg, 55.4 years for IS). NPP was the largest occupational group (32.5%), followed by industry (20.8%), and NDT(15.1%). NPP workers accounted for approximately half of stroke cases. Until diagnosis or end of follow-up, the mean employment duration was 4.1 years, and 34.5% of the population had worked for <1 year. The mean heart dose was 4.1 mGy (max 992.6 mGy), slightly higher than the mean brain dose of 3.9 mGy (max 954.9 mGy). The values were higher in disease groups: the mean radiation dose range was 4.7–5.7 mGy.

**Table 2:** Demographic and occupational characteristics of 186 233 Korean radiation workers by subtypes of circulatory diseases. [IS=ischemic stroke; HS=hemorrhagic stroke; IHD=ischemic heart disease; AMI=acute myocardial infarction; VHD=valvular heart disease; HF= heart failure]

		Total (N=186 233) ^a^		IS (N=1888) ^b^		HS (N=775) ^c^		IHD (N=8562) ^d^		AMI (N=1713) ^e^		VHD (N=788) ^f^		HF (N=5892) ^g^
		N (%)		N (%)		N (%)		N (%)		N (%)		N (%)		N (%)
Population at risk		186 233 (100.0)		186 030 (100.0)		186 079 (100.0)		184 522 (100.0)		185 908 (100.0)		185 966 (100.0)		185 559 (100.0)
Sex														
	Male	155 342 (83.4)		1777 (94.1)		730 (94.2)		8092 (94.5)		1672 (97.6)		698 (88.6)		5387 (91.4)
	Female	30 891 (16.6)		111 (5.9)		45 (5.8)		470 (5.5)		41 (2.4)		90 (11.4)		505 (8.6)
Attained age (years)														
	<30	17 269 (9.3)		18 (1.0)		20 (2.6)		252 (2.9)		27 (1.6)		40 (5.1)		202 (3.4)
	30–39	44 981 (24.2)		154 (8.2)		109 (14.1)		1252 (14.6)		199 (11.6)		133 (16.9)		831 (14.1)
	40–49	55 876 (30.0)		443 (23.5)		252 (32.5)		2563 (29.9)		500 (29.2)		189 (24.0)		1516 (25.7)
	50–59	41 718 (22.4)		575 (30.5)		242 (31.2)		2757 (32.2)		572 (33.4)		201 (25.5)		1719 (29.2)
	60–69	21 398 (11.5)		445 (23.6)		111 (14.3)		1366 (16.0)		308 (18.0)		148 (18.8)		1165 (19.8)
	≥70	4991 (2.7)		253 (13.4)		41 (5.3)		372 (4.3)		107 (6.3)		77 (9.8)		459 (7.8)
Year of birth														
	Before 1960	20 938 (11.2)		995 (52.7)		267 (34.5)		3536 (41.3)		747 (43.6)		333 (42.3)		2213 (37.6)
	1960s	37 339 (20.0)		541 (28.7)		291 (37.6)		2720 (31.8)		586 (34.2)		204 (25.9)		1725 (29.3)
	1970s	54 020 (29.0)		270 (14.3)		157 (20.3)		1681 (19.6)		289 (16.9)		169 (21.5)		1248 (21.2)
	1980s	49 853 (26.8)		75 (4.0)		54 (7.0)		535 (6.3)		80 (4.7)		68 (8.6)		582 (9.9)
	After 1990	24 083 (12.9)		7 (0.4)		6 (0.8)		90 (1.1)		11 (0.6)		14 (1.8)		124 (2.1)
Facility														
	Public	3986 (2.1)		36 (1.9)		14 (1.8)		215 (2.5)		34 (2.0)		15 (1.9)		143 (2.4)
	Education	27 623 (14.8)		99 (5.2)		47 (6.1)		625 (7.3)		72 (4.2)		84 (10.7)		482 (8.2)
	Military	847 (0.5)		6 (0.3)		2 (0.3)		13 (0.2)		5 (0.3)		4 (0.5)		16 (0.3)
	Non-destructive testing	28 122 (15.1)		160 (8.5)		100 (12.9)		808 (9.4)		172 (10.0)		65 (8.3)		572 (9.7)
	Industry	38 663 (20.8)		434 (23.0)		184 (23.7)		1973 (23.0)		429 (25.0)		150 (19.0)		1272 (21.6)
	Research	8397 (4.5)		60 (3.2)		22 (2.8)		446 (5.2)		63 (3.7)		46 (5.8)		272 (4.6)
	Nuclear power plants	60 523 (32.5)		976 (51.7)		362 (46.7)		3878 (45.3)		841 (49.1)		356 (45.2)		2549 (43.3)
	Medical	18 072 (9.7)		117 (6.2)		44 (5.7)		604 (7.1)		97 (5.7)		68 (8.6)		586 (10.0)
Employment duration until disease or follow-up end (years)														
	<1	64 271 (34.5)		815 (43.2)		336 (43.4)		3167 (37.0)		649 (37.9)		295 (37.4)		2243 (38.1)
	1–4	75 754 (40.7)		620 (32.8)		246 (31.7)		3083 (36.0)		633 (37.0)		276 (35.0)		2128 (36.1)
	5–9	23 915 (12.8)		198 (10.5)		94 (12.1)		971 (11.3)		190 (11.1)		89 (11.3)		676 (11.5)
	10–19	14 740 (7.9)		179 (9.5)		66 (8.5)		917 (10.7)		165 (9.6)		90 (11.4)		546 (9.3)
	≥20	7553 (4.1)		76 (4.0)		33 (4.3)		424 (5.0)		76 (4.4)		38 (4.8)		299 (5.1)
Cumulative heart dose (mGy)														
	0	65 630 (35.2)		527 (27.9)		193 (24.9)		2246 (26.2)		465 (27.2)		229 (29.1)		1683 (28.6)
	>0–1	53 538 (28.7)		481 (25.5)		219 (28.3)		2325 (27.2)		427 (24.9)		209 (26.5)		1616 (27.4)
	>1.0–2.5	21 583 (11.6)		233 (12.3)		109 (14.1)		1196 (14.0)		253 (14.8)		113 (14.3)		809 (13.7)
	>2.5–5.0	14 860 (8.0)		212 (11.2)		61 (7.9)		879 (10.3)		171 (10.0)		75 (9.5)		559 (9.5)
	>5.0–10.0	12 403 (6.7)		202 (10.7)		87 (11.2)		798 (9.3)		169 (9.9)		69 (8.8)		501 (8.5)
	>10.0–20.0	8658 (4.6)		106 (5.6)		48 (6.2)		511 (6.0)		97 (5.7)		36 (4.6)		352 (6.0)
	>20.0–50.0	6749 (3.6)		83 (4.4)		39 (5.0)		407 (4.8)		95 (5.6)		44 (5.6)		262 (4.5)
	> 50.0	2812 (1.5)		44 (2.3)		19 (2.5)		200 (2.3)		36 (2.1)		13 (1.7)		110 (1.9)
Cumulative brain dose (mGy)														
	0	65 630 (35.2)		527 (27.9)		193 (24.9)		2246 (26.2)				229 (29.1)		1683 (28.6)
	>0–1	55 220 (29.7)		494 (26.2)		223 (28.8)		2389 (27.9)		444 (25.9)		216 (27.4)		1716 (28.7)
	>1.0–2.5	21 293 (11.4)		235 (12.5)		113 (14.6)		1218 (14.2)		255 (14.9)		115 (14.6)		817 (13.7)
	>2.5–5.0	14 666 (7.9)		218 (11.6)		61 (7.9)		878 (10.3)		171 (10.0)		75 (9.5)		580 (9.7)
	>5.0–10.0	12 073 (6.5)		198 (10.5)		81 (10.5)		776 (9.1)		165 (9.6)		65 (8.3)		480 (8.0)
	>10.0–20.0	8237 (4.4)		96 (5.1)		48 (6.2)		476 (5.6)		90 (5.3)		33 (4.2)		330 (5.5)
	>20.0–50.0	6542 (3.5)		79 (4.2)		39 (5.0)		392 (4.6)		89 (5.2)		42 (5.3)		253 (4.2)
	>50.0	2572 (1.4)		41 (2.2)		17 (2.2)		187 (2.2)		34 (2.0)		13 (1.7)		104 (1.7)

^a^ PY: mean (SD)=13.3 (6.4), sum=2 483 113. Attained age: mean (SD)=45.6 (12.1). Year of birth: mean (SD)=1975.3 (12.3). Employment duration until disease or follow-up end: mean (SD)=4.1 (6.1). Cumulative heart dose: mean (SD)=4.1 (13.3) range=0–992.6. Cumulative brain dose: mean (SD)=3.9 (12.9) range=0–954.9. ^b^ PY: mean (SD)=12.9 (6.1), sum=2 402 411. Attained age: mean (SD)=55.4 (12.0). Year of birth: mean (SD)=1958.7 (12.1). Employment duration until disease or follow-up end: mean (SD)=4.0 (6.0). Cumulative heart dose: mean (SD)=5.5 (14.4) range=0–215.4. Cumulative brain dose: mean (SD)=5.2 (14.2) range=0–218.1. ^c^ PY: mean (SD)=13.0 (6.1), sum=2 409 224. Attained age: mean (SD)=50.2 (11.4). Year of birth: mean (SD)=1963.0 (11.4). Employment duration until disease or follow-up end: mean (SD)=4.1 (6.3). Cumulative heart dose: mean (SD)=5.7 (13.5) range=0–103.6. Cumulative brain dose: mean (SD)=5.4 (13.2) range=0–104.9. ^d^ PY: mean (SD)=12.6 (6.1), sum=2 329 891. Attained age: mean (SD)=50.2 (11.1). Year of birth: mean (SD)=1962.4 (11.6). Employment duration until disease or follow-up end: mean (SD)=4.6 (6.5). Cumulative heart dose: mean (SD)=5.7 (15.8) range=0–299.6. Cumulative brain dose: mean (SD)=5.5 (15.6) range=0–303.4. ^e^ PY: mean (SD)=12.9 (6.1), sum=2 400 113. Attained age: mean (SD)=52.0 (11.2). Year of birth: mean (SD)=1961.1 (11.4). Employment duration until disease or follow-up end: mean (SD)=4.3 (6.2). Cumulative heart dose: mean (SD)=5.7 (15.3) range=0–299.6. Cumulative brain dose: mean (SD)=5.5 (15.1) range=0–303.4. ^f^ PY: mean (SD)=12.9 (6.1), sum=2 407 074. Attained age: mean (SD)=51.2 (13.6). Year of birth: mean (SD)=1962.4 (13.3). Employment duration until disease or follow-up end: mean (SD)=4.6 (6.6). Cumulative heart dose: mean (SD)=5.2 (14.4) range=0–170.2. Cumulative brain dose: mean (SD)=5.0 (14.4) range=0–172.3. ^g^ PY: mean (SD)=12.8 (6.1), sum=2 380 843. Attained age: mean (SD)=51.8 (12.4). Year of birth: mean (SD)=1963.9 (12.6). Employment duration until disease or follow-up end: mean (SD)= 4.4 (6.5). Cumulative heart dose: mean (SD)=5.0 (13.4) range=0–193.4. Cumulative brain dose: mean (SD)=4.7 (13.2) range=0–195.9.

### Non-radiation risk factors

Supplementary table S2 summarizes distribution of insurance contribution levels and non-radiation risk factors. Approximately 50% of participants were in the high-income group, whereas <20% were in the low-income group. Among all participants, 37.1% were current smokers, and 30% were overweight or obese. These proportions were notably higher among those with IHD (44.1% smokers) and AMI (48.1% smokers), and >40% were overweight or obese. High BP or elevated fasting glucose levels was 2–3 times more prevalent in the disease groups than the overall population.

### Incidence rates of circulatory disease subtypes

Table 3 presents incidence rates per 100 000 person-years and SIR, stratified by sex. IHD had the highest incidence rate (367.6 cases per 100 000 person-years), followed by HF (247.5 cases). The incidence rate of IS (78.6 cases) was more than twice that of HS (32.2 cases). Overall, the incidence rate was 2–3 times higher among males than females. Notably, the incidence of AMI among males was approximately 7 times higher than among females. Most diseases had significantly lower SIR (0.70–0.96). However, the SIR for VHD was not statistically significantly different from that of the general population.

**Table 3:** Incidence rate (IR) per 100 000 person-years and sex- and age- standardized incidence ratio (SIR) with 95% confidence interval (CI) for circulatory disease subtypes by sex. [IS=ischemic stroke; HS=hemorrhagic stroke; IHD=ischemic heart disease; AMI=acute myocardial infarction; VHD=valvular heart disease; HF=heart failure; PY=person-years].

	Total					Males					Females			
	Observed cases ^a^	PY	IR	SIR (95% CI)		Observed cases ^a^	PY	IR	SIR (95% CI)		Observed cases ^a^	PY	IR	SIR (95% CI)
IS	1888	2 401 900	78.6	0.73 (0.70–0.77)		1777	2 042 610	87.0	0.73 (0.69–0.77)		111	359 286	30.9	0.86 (0.70–1.07)
HS	775	2 408 710	32.2	0.70 (0.65–0.75)		730	2 049 040	35.6	0.70 (0.65–0.76)		45	359 669	12.5	0.62 (0.45–0.85)
IHD	8562	2 329 390	367.6	0.96 (0.93–0.98)		8092	1 973 740	410.0	0.97 (0.94–0.99)		470	355 641	132.2	0.78 (0.70–0.86)
AMI	1713	2 399 600	71.4	0.90 (0.85–0.95)		1672	2 040 010	82.0	0.91 (0.86–0.96)		41	359 593	11.4	0.63 (0.45–0.90)
VHD	788	2 406 560	32.7	0.98 (0.91–1.06)		698	2 047 370	34.1	1.01(0.93–1.10)		90	359 194	25.1	0.77 (0.60–0.99)
HF	5892	2 380 330	247.5	0.89 (0.86–0.91)		5387	2 023 360	266.2	0.89 (0.86–0.92)		505	356 973	141.5	0.83 (0.74–0.93)

^a^ Patients from 2002 to 2019 were included in the analysis.

### Dose–response relationship

Table 4 presents RR of circulatory disease by dose groups. Compared with the zero-dose group, most circulatory disease subtypes had RR <1 across dose categories, indicating no increased risk. Although RR >1 were observed for HS and VHD in some dose groups, they were not statistically significant. Table 5 shows ERR estimates per 10 mGy. Overall, no clear evidence of a positive dose–response relationship was observed. With a 10-year lag, the ERR estimate for HS was positive (ERR 0.014, 95% CI -0.049–0.077), while other subtypes showed negative estimates. Notably, a statistically significant negative ERR was observed for HF. In analyses restricted to workers employed >1 year, ERR estimates increased slightly across subtypes but remained non-significant. ERR among those first monitored in 1984 tended to be higher (eg, HS: 0.088, 95% CI -0.085‒0.262; IS: 0.033, 95% CI -0.045‒0.110), though still not statistically significant. Among those first monitored from 2002, ERR for IS and HS showed opposite directions compared to the full cohort. Additional analyses grouping stroke and heart disease outcomes by organ-specific absorbed dose yielded similar trends to the subtype-specific analyses, with no evidence of increased risk (supplementary table S3). In addition to the linear ERR model, we evaluated a linear-quadratic (LQ) model to assess the potential non-linearity in the dose–response relationship. As shown in supplementary table S4, the model fit was significantly improved for IHD (P=0.028) and HF (P=0.035) under the LQ model compared with the linear model.

**Table 4:** Relative rate (RR) ^a^ and 95% confidence interval (CI) by dose (mGy) group for circulatory disease incidence. [IS=ischemic stroke, HS=hemorrhagic stroke; IHD=ischemic heart disease; AMI=acute myocardial infarction; VHD=valvular heart disease; HF=heart failure].

Dose group		IS				HS				IHD				AMI				VHD				HF		
		Cases	RR	95% CI		Cases	RR	95% CI		Cases	RR	95% CI		Cases	RR	95% CI		Cases	RR	95% CI		Cases	RR	95% CI
Without lag																								
	0	527	1			193	1			2240	1			465	1			229	1			1678	1	
	≤1	494	0.87	0.77–0.98		223	0.98	0.81–1.19		2333	0.91	0.86–0.97		428	0.84	0.74–0.96		209	0.81	0.67–0.98		1624	0.91	0.85–0.98
	>1–2.5	235	0.83	0.71–0.97		113	1.00	0.79–1.26		1206	0.92	0.86–0.99		255	0.97	0.83–1.13		113	0.91	0.72–1.14		807	0.94	0.86–1.02
	>2.5–5.0	218	0.99	0.84–1.16		62	0.72	0.54–0.95		869	0.86	0.79–0.93		170	0.82	0.68–0.97		76	0.81	0.62–1.05		559	0.85	0.77–0.94
	>5.0–10.0	200	1.01	0.86–1.19		80	1.06	0.82–1.38		806	0.89	0.82–0.96		170	0.89	0.75–1.07		68	0.82	0.62–1.07		501	0.86	0.78–0.95
	>10.0–20.0	93	0.76	0.61–0.94		48	1.00	0.73–1.38		503	0.83	0.75–0.91		97	0.76	0.61–0.95		37	0.66	0.47–0.94		352	0.90	0.80–1.01
	>20.0–50.0	80	0.95	0.75–1.20		39	1.10	0.78–1.56		408	0.96	0.86–1.06		92	1.03	0.82–1.29		43	1.14	0.82–1.58		261	0.94	0.82–1.07
	>50.0	41	0.94	0.69–1.30		17	1.00	0.61–1.64		197	0.93	0.80–1.07		36	0.79	0.56–1.11		13	0.72	0.41–1.26		110	0.81	0.67–0.98
5-year lagged																								
	0	600	1			237	1			2756	1			547	1			268	1			1954	1	
	≤1	454	0.83	0.73–0.94		198	0.90	0.74–1.09		2114	0.87	0.82–0.92		397	0.82	0.72–0.94		191	0.83	0.69–1.01		1525	0.90	0.84–0.97
	>1–2.5	235	0.85	0.73–0.99		104	0.94	0.75–1.19		1142	0.91	0.85–0.97		245	0.97	0.83–1.13		101	0.90	0.71–1.14		734	0.90	0.82–0.97
	>2.5–5.0	208	0.96	0.82–1.13		58	0.69	0.51–0.92		813	0.83	0.77–0.90		163	0.81	0.68–0.97		80	0.94	0.73–1.20		518	0.82	0.74–0.90
	>5.0–10.0	191	0.99	0.84–1.17		83	1.15	0.89–1.48		740	0.85	0.78–0.92		154	0.85	0.71–1.02		64	0.85	0.65–1.12		503	0.90	0.82–1.00
	>10.0–20.0	92	0.78	0.63–0.98		44	0.98	0.71–1.35		464	0.82	0.74–0.91		91	0.77	0.61–0.96		37	0.75	0.53–1.06		327	0.88	0.78–0.99
	>20.0–50.0	72	0.90	0.71–1.15		36	1.09	0.77–1.55		353	0.89	0.80–1.00		82	0.99	0.78–1.25		37	1.12	0.79–1.59		236	0.91	0.79–1.04
	>50.0	36	0.89	0.64–1.25		15	0.97	0.57–1.64		180	0.94	0.81–1.10		34	0.84	0.59–1.18		10	0.65	0.35–1.23		95	0.77	0.62–0.94
10-year lagged																								
	0	739	1			312	1			3546	1			695	1			339	1			2421	1	
	≤1	406	0.81	0.72–0.92		165	0.85	0.70–1.03		1823	0.88	0.83–0.93		347	0.82	0.72–0.94		163	0.85	0.70–1.03		1306	0.88	0.82–0.95
	>1–2.5	210	0.80	0.68–0.93		95	0.93	0.74–1.18		958	0.86	0.80–0.92		211	0.92	0.78–1.07		92	0.94	0.74–1.19		679	0.90	0.82–0.98
	>2.5–5.0	185	0.90	0.76–1.06		57	0.73	0.55–0.97		744	0.85	0.79–0.92		145	0.79	0.66–0.95		68	0.90	0.69–1.17		489	0.84	0.76–0.93
	>5.0–10.0	172	0.96	0.82–1.14		62	0.96	0.73–1.27		653	0.87	0.80–0.95		143	0.89	0.74–1.07		52	0.80	0.59–1.07		435	0.86	0.77–0.95
	>10.0–20.0	82	0.78	0.62–0.98		38	0.98	0.69–1.37		386	0.82	0.73–0.91		74	0.72	0.56–0.91		39	0.94	0.67–1.31		284	0.86	0.76–0.98
	>20.0–50.0	66	0.92	0.71–1.18		35	1.21	0.85–1.72		311	0.93	0.82–1.04		70	0.96	0.75–1.23		26	0.94	0.63–1.41		194	0.85	0.74–0.99
	>50.0	28	0.79	0.54–1.16		11	0.85	0.46–1.55		141	0.92	0.77–1.08		28	0.83	0.57–1.21		9	0.72	0.37–1.40		84	0.80	0.65–1.00

^a^ Adjusted for sex, attained age (categorical), calendar year (categorical), and birth year (categorical).

**Table 5:** Excess relative risk (ERR) ^a^ per 10 mGy ^b^ and 95% confidence interval (CI) for circulatory disease incidence by subtypes. [IS=ischemic stroke; HS=hemorrhagic stroke; IHD=ischemic heart disease; AMI=acute myocardial infarction; VHD=valvular heart disease; HF=heart failure.]

			IS			HS			IHD			AMI			VHD			HF	
			ERR	95% CI		ERR	95% CI		ERR	95% CI		ERR	95% CI		ERR	95% CI		ERR	95% CI
Without lag ^a^			-0.004	-0.035–0.027		0.010	-0.042–0.062		-0.005	-0.019–0.010		-0.012	-0.042–0.019		-0.008	-0.057–0.042		-0.014	-0.031–0.003
5-year lagged ^a^			-0.008	-0.041–0.024		0.014	-0.043–0.070		-0.005	-0.020–0.010		-0.010	-0.043–0.023		-0.015	-0.066–0.037		-0.018	-0.036– -0.001
10-year lagged ^a^			-0.017	-0.050–0.017		0.014	-0.049–0.077		-0.008	-0.025–0.009		-0.013	-0.049–0.023		-0.019	-0.075–0.037		-0.020	-0.039– -0.001
	Adjusted for duration of working ^c^		0.168	0.081–0.255		0.280	0.112–0.447		0.081	0.050–0.111		0.120	0.043–0.198		0.035	-0.052–0.123		0.044	0.012–0.077
	Adjusted further ^d^		-0.016	-0.050–0.019		0.021	-0.048–0.089		-0.008	-0.025–0.009		-0.017	-0.052–0.018		-0.001	-0.061–0.059		-0.017	-0.036–0.003
	Working duration >1 year ^a^		0.023	-0.022–0.068		0.068	-0.017–0.153		0.008	-0.012–0.027		0.002	-0.039–0.043		-0.008	-0.069–0.054		-0.007	-0.029–0.015
	Year of first dose record ^a^																		
		1984	0.033	-0.045–0.110		0.088	-0.085–0.262		-0.003	-0.034–0.028		0.003	-0.073–0.078		-0.045	-0.115–0.025		0.002	-0.038–0.042
		≥1985	-0.031	-0.072–0.011		-0.011	-0.081–0.060		-0.008	-0.030–0.014		-0.005	-0.052–0.043		-0.022	-0.095–0.051		-0.029	-0.052– -0.006
		≥2002	0.132	-0.239–0.503		-0.023	-0.467–0.422		-0.104	-0.200– -0.008		-0.090	-0.321–0.142		-0.107	-0.424–0.211		-0.032	-0.149–0.085
	Restricted dose ^a^ (mGy)																		
		<200	-0.014	-0.051–0.023		0.023	-0.047–0.092		-0.008	-0.027–0.010		-0.013	-0.052–0.025		-0.018	-0.078–0.042		-0.019	-0.040–0.002
		<100	-0.011	-0.059–0.037		0.048	-0.042–0.137		-0.009	-0.032–0.014		0.001	-0.050–0.052		-0.033	-0.105–0.040		-0.022	-0.048–0.004
		<50	-0.029	-0.103–0.046		0.067	-0.070–0.204		-0.032	-0.067–0.003		-0.010	-0.088–0.069		-0.009	-0.133–0.114		-0.039	-0.080–0.002
		<20	-0.051	-0.187–0.085		0.021	-0.217–0.258		-0.140	-0.201– -0.080		-0.172	-0.299– -0.045		-0.162	-0.361–0.036		-0.097	-0.171– -0.022

^a^ Estimates based on linear model, adjusted for sex, attained age, calendar year, and birth year. ^b^ Brain dose was used for IS and HS, while heart dose was used for the other cardiac outcomes. ^c^ Adjusted for sex, attained age, calendar year, birth year, and working duration. ^d^ Adjusted for sex, attained age, calendar year, birth year, income, smoking, blood pressure, blood glucose level, and BMI.

## Discussion

We evaluated the risk of circulatory disease subtypes associated with low-dose occupational radiation exposure. The ERR estimates for most circulatory disease subtypes did not show a consistent positive association with radiation dose. The risk coefficients remained negative for most subtypes, except for HS, which showed a positive but non-significant estimate.

Some occupational radiation studies have revealed negative ERR estimates or null associations (22, 23), which may have been partly influenced by methodological limitations rather than indicating a true protective effect of radiation. Potential sources of bias include workforce selection (24), exposure misclassification (25), and incomplete exposure or outcome data (13). One possible bias is the healthy worker survivor effect, where healthier workers tend to remain employed longer, accumulate higher radiation doses, and exhibit lower disease risks compared with short-term workers, who may leave employment due to health-related issues (24, 26). In the present cohort, workers employed for <1 year had a mean heart dose of 0.87 mGy, with ERR estimates per 10 mGy of 0.34 (95% CI -0.13–0.80) for IS and 0.40 (95% CI -0.29–1.08) for HS (supplementary table S5). In contrast, those employed for ≥1 had a higher mean dose of 5.81 mGy, yet their ERR estimates were low at 0.02 (95% CI -0.02–0.07) for IS and 0.07 (95% CI -0.02–0.15) for HS. In addition, across all subtypes, workers employed for <1 year showed higher SIR than those employed for ≥1 year. Notably, the SIR for IHD among short-term workers was significantly elevated (SIR 1.07, 95% CI 1.03–1.11). These findings suggest that short-term workers exhibited higher health risks despite their lower radiation exposure, consistent with the healthy worker survivor effect. Furthermore, when restricting the analysis to workers employed for ≥1 year, the previously negative ERR estimates for major circulatory diseases (IS, IHD, and AMI) shifted to positive values, although they remained statistically nonsignificant.

In a previous study (15), IHD showed a significant negative association, which may have been partially influenced by outcome misclassification. In the current study, using the same dataset, a more refined and specific case definition for IHD was employed, and the previously observed negative association disappeared. Minimizing outcome misclassification is crucial in radiation epidemiology, where risk estimates are often small in magnitude and highly susceptible to bias (13). The use of broad disease categories without clear operational definitions may inadvertently include heterogeneous conditions with differing etiologies or non-radiogenic profiles. Such aggregation can obscure true effects or result in “false negative” findings and is of particular concern in studies reporting negligible association between exposure and disease (27), as combining disorders with varying radiosensitivity can distort the observed risk estimates. One notable example in the current study is HF; no refined operational definition was applied, and a significantly decreased ERR was observed, which may also reflect, at least in part, outcome misclassification. HF is particularly prone to outcome misclassification because it encompasses a wide range of clinical presentations and underlying causes and is often recorded as the cause of death even though it frequently reflects an intermediate rather than underlying cause (28). To further explore the shape of the dose–response relationship, we also examined a linear-quadratic model. For IHD and HF, the LQ model demonstrated significantly better fit compared to the linear model, suggesting a potential non-linear pattern of association. However, given the limited dose variability in our data, the findings should be interpreted with caution and confirmed in future studies with greater exposure contrast.

Furthermore, we employed a passive follow-up method by linking disease and dose registries. In addition, some exposures occurred before the establishment of a national disease database capable of supporting systematic follow-up of incidence, potentially resulting in loss to follow-up (13). This is particularly relevant for earlier workers with higher cumulative doses, whose disease incidence may have been incompletely captured, potentially leading to underestimation of risk and attenuation of dose–response relationships. When the analysis was restricted to workers first monitored for dose after 2002, when the NHID was established, lower ERR were observed for most circulatory disease subtypes (except IS) compared with the full cohort. These findings suggest that incomplete follow-up may have had only a limited influence on the overall lack of positive associations. Nevertheless, this subgroup represents a relatively recent cohort with lower cumulative doses, younger attained ages, and, consequently, a lower expected number of circulatory disease cases. Given that this group benefits from improved dosimetric accuracy and complete follow-up of both exposure and outcome data, continued follow-up is warranted to obtain more robust and reliable risk estimates.

The validity and completeness of historical dose records remain critical methodological concerns in occupational radiation studies (29, 30). Early dosimetry was limited by missing data and the imprecision of film badge technology. In this study, workers first monitored in 1984, the year a systematic dosimetry system was established, tended to show higher ERR estimates, which may reflect their greater cumulative doses and attained age, and longer follow-up. Alternatively, the elevated ERR may be influenced by earlier dose underestimation, leading to inflated risk coefficients. Consistent with these findings, a Canadian study revealed increased solid cancer mortality among workers first monitored between 1956 and 1964 (31), and early Sellafield workers demonstrated elevated ERR for circulatory disease mortality (32). However, some studies have reported contrasting findings. For example, early Mayak workers showed lower relative rates despite higher doses, possibly due to dose underestimation or other early workplace hazards (32). A recent study of the INWORKS (25) revealed higher ERR estimates for solid cancer among workers employed in more recent calendar periods relative to the full cohort. Although these workers generally had lower cumulative doses, their risk estimates were less likely to be susceptible to bias from dose misclassification (25). However, if radiation were a major causal factor, radiation-related effects would be expected to be detectable in the earlier-hired subcohort with higher doses. These complexities underscore the need for further investigation into temporal variations in dose uncertainty across hiring periods and the shape of the dose–response relationship in the low-dose range (33).

In addition to uncertainties in historical dose estimates, the validity of record linkage between occupational and health databases represents another potential methodological concern in cohort studies. In this study, the linkage between the radiation worker cohort and the national health insurance database was conducted using each individual’s unique resident registration number, a permanent personal identifier assigned to all Korean citizens. This deterministic linkage was performed by the Big Data Office of the NHIS, an authorized agency with legal access to personal identifiers. Given the use of a unique identifier and centralized linkage by a trusted third party, the likelihood of linkage error was minimal. Furthermore, because the linkage was applied to the entire enumerated cohort of monitored radiation workers—regardless of exposure level or health status—the probability of differential linkage according to occupational radiation exposure is likely to be minimal. Any linkage-related misclassification, if present, would be expected to be non-differential with respect to dose, thereby biasing the estimates toward the null rather than inflating them.

The mean cumulative heart dose in our cohort was 4.11 mGy, which is lower than that reported for most other occupational groups (eg, 25 mSv in INWORKS, 47 mSv among uranium miners, and 430 mGy among Mayak workers) (10, 34, 35). Correspondingly, the ERR per Gy was -1.7 for IS and -0.8 for IHD, with neither estimate reaching statistical significance. Although the ERR per Gy for HS was positive (1.4), it was not statistically significant. These values contrast with those of the Life Span Study (ERR per Gy=0.09 for stroke, 0.14 for heart disease) and with INWORKS (0.17 for IHD, 0.49 for CeVD) (10, 36), suggesting either a genuinely weaker dose–response at very low cumulative doses or limited statistical power in our cohort. Because most previous worker studies have relied on mortality, direct incidence–mortality comparisons are rare; however, the Mayak investigations—where both outcomes were analyzed—show that ERR for CeVD incidence consistently exceed those for mortality, implying that incidence data capture a broader spectrum of radiation-associated disease (35, 37). Intensive surveillance and the resulting diagnosis of milder cases may partly explain this gap, underscoring the importance of incorporating both incidence and mortality when assessing circulatory risks from low-dose ionizing radiation (38).

The higher risk coefficient for HS compared with IS is consistent with findings from both the LSS and NRRW cohorts, where a suggestive increase in risk was observed for HS but not for IS (39, 40). These differences may reflect heterogeneity in the pathophysiological mechanisms underlying each subtype (37, 39, 40). HS is more strongly influenced by elevated BP than IS and is often linked to fibrinoid necrosis of small vessels, a process initiated by proinflammatory cytokines. In atomic bomb survivors, hypertension and inflammation may further contribute to this pathology, increasing susceptibility to HS. A study of Russian Mayak nuclear workers revealed a significantly increased ERR per Gy for overall CeVD but not for total, HS, or IS (37). This discrepancy may be partly explained by differences in underlying mechanisms. Chronic CeVD is primarily driven by progressive atherosclerosis of cerebral vessels, a multifactorial process that can be accelerated by ionizing radiation (6). In contrast, acute CeVD, such as stroke, are more commonly caused by atherothrombosis, embolism, or hypertension, which may be less directly influenced by radiation exposure. In another study of Mayak workers, significant positive dose–response relationships were reported for several heart disease subtypes, including IHD, HF, and angina pectoris (35). In contrast, we observed non-significant negative ERR for heart disease outcomes, with no notable subtype-specific patterns. These differences may reflect variations in cohort characteristics, dose distributions, or diagnostic practices across study populations.

VHD is a recognized complication of high-dose radiation exposure (5). Unlike age-related valvular calcification, radiation-induced valvular damage is characterized by greater fibrosis and lesser calcification. These pathological differences suggest that the mechanisms underlying radiation-related valvulopathy may differ from those of degenerative disease (5). In our study, we assessed the risk of VHD but found no significant association, likely because of the relatively low radiation exposure levels in our cohort. Given the subtle and long-latency nature of health effects from low-dose radiation exposure, traditional endpoints such as mortality or clinically diagnosed disease may be insufficient to detect early biological changes (41). Future research may benefit from integrating multiomics approaches, including genomic, proteomic, and metabolomic data, with traditional cohort studies. Such integration could improve the detection of biomarkers and biological pathways associated with radiation-induced cardiovascular disease, thereby enhancing risk assessment and the understanding of low-dose effects.

The overall incidence rate of circulatory diseases among radiation workers was statistically significantly lower than that of the general population, indicating the presence of the healthy worker effect. This effect has been observed in other nuclear worker cohorts (22, 42). In the current study, the SIR for stroke (≈0.7) was lower than that of heart disease (>0.9), consistent with previous reviews indicating that the magnitude of the healthy worker effect may vary by disease (43). This variation may be explained by differences in disease severity and their impact on continued employment. Diseases such as certain heart conditions may allow individuals to remain employed after diagnosis if their disease is manageable, thereby reducing the magnitude of the effect. In contrast, stroke often leads to sudden disability and early workforce exit, resulting in a more pronounced healthy worker effect. Previous studies on Korean radiation diagnostic workers also revealed that the SMR for CeVD is lower than that for IHD (44, 45). Meanwhile, the SIR for VHD was close to 1, indicating no significant difference from the general population. This may be due to its degenerative nature and the difficulty of predicting its onset at the time of employment.

The timing of radiation-related health effects may vary based on the disease. According to previous studies, applying longer lag periods of 15 or 20 years for the circulatory disease tended to result in higher risk estimates compared to 5- or 10-year lags; however, these differences were not statistically significant (9). In our study, the risk estimates were also similar between the 5- and 10-year lag periods. With longer follow-up in the future, it may be possible to explore a wider range of lag periods.

This study has several notable strengths. First, it leverages the Korean Radiation Workers Study, one of the largest nationwide occupational cohorts linking individual dosimetry records with comprehensive administrative health data, providing more than 2.4 million person-years of follow-up. Second, the availability of health-screening information allowed adjustment for key confounders rarely captured in radiation-worker studies. Third, we applied refined, clinically validated operational definitions for six circulatory-disease subtypes, enabling subtype-specific risk estimation and reducing outcome misclassification. Finally, by analyzing incidence rather than mortality alone, the study captures a wider spectrum of radiation-associated disease and facilitates latency assessments. Nevertheless, certain limitations warrant caution. Statistical power remains limited at the very low cumulative doses observed (mean heart dose ≈ 4 mGy), and CI are consequently wide. Historical dose records prior to 1984 are incomplete, which may lead to exposure misclassification and potentially bias observed dose–response trends. The lack of outcome data prior to 2002 may have led to under ascertainment of earlier cases. Although our subgroup analysis among workers with complete follow-up since 2002 showed broadly consistent results, this limitation may still have introduced some bias in the estimation of dose–response relationships. Although we excluded employees with <1 year of service and conducted stratified analyses, healthy-worker survivor bias cannot be completely ruled out. Future analyses will consider applying g-methods to better control for time-varying confounding and dynamic selection mechanisms. Although the current analysis adjusted for several known confounders such as smoking status and BMI using their most recent values, residual confounding may persist due to temporal changes in these factors and the lack of information on unmeasured variables such as detailed occupational co-exposures and shift work. Future work incorporating fully time-updated covariates is warranted. Finally, the cohort’s relatively short employment duration and young attained ages limit our ability to evaluate very long-latency circulatory outcomes. Continued follow-up, along with the potential incorporation of biomarker may help address these limitations and further elucidate the dose–response relationship in the low-dose range.

In conclusion, no clear positive association was observed between low-dose occupational radiation exposure and major circulatory disease subtypes. However, the variability in subtype-specific SIR and ERR estimates suggests potential heterogeneity in radiation-related risk, highlighting the importance of continued monitoring of radiation workers, particularly for subtype-specific effects. These results underscore the need for enhanced disease classification, improved dose assessment, and longer follow-up to better characterize potential risks. Importantly, the implications extend to future radiation protection policy and health surveillance strategies, especially in occupational settings involving chronic low-dose exposure.

## Supplementary material

Supplementary material
