# Supplementary material for: Risk of incident stroke and heart disease subtypes in a nationwide cohort of Korean radiation workers
Source: Scand J Work Environ Health. 2025 Oct 30;51(6):537–49. doi: 10.5271/sjweh.4251 (PMC12593710; doi:10.5271/sjweh.4251)
Supplement: Supplementary material [file SJWEH-51-537-S001.pdf]

# Risk of incident stroke and heart disease subtypes in a nationwide cohort of Korean radiation workers[1]

*by Eun Shil Cha, PhD, Kyunghee Chae, PhD, Haesu Jeong, MS, Dalnim Lee, PhD, Soojin Park, PhD, Ga Bin Lee, PhD, Minsu Cho, MD, PhD, Hyeonseok Nam, MD, Songwon Seo, PhD* [2]

1. Supplementary material
2. Correspondence to: Songwon Seo, National Radiation Emergency Medical Center, Korea Institute of Radiological and Medical Sciences, 75 Nowon-gil, Nowon-gu, Seoul, 01812, Republic of Korea. [E-mail: seo@kirams.re.kr]

Supplementary Table S1. Procedure and diagnostic insurance claim codes related to stroke and IHD/AMI

| Diagnostic test or procedures | Insurance claim code |
|---|---|
| Stroke-related imaging tests | (CT) HA441, HA451, HA461, HA511, HA521, HA531, HA551, HA561, HA471, HA851<br>(MRI) HE101, HE102, HE135, HE136, HE501, HE502, HE535, HE201, HE235, HE236, HE301, HE302, HE401, HE202, HE402, HF101, HF102, HF201, HF202, HF103, HF104, HF105, HF106, HF107, HF203, HF305, HF306, HI101, HI135, HI201, HI235, HI301, HI401, HI501, HI535, HJ101, HJ135, HJ201, HJ235, HJ301, HJ401, HJ501, HJ535, HJ601, HJ635, HJ701, HJ735<br>(Cerebral angiography) HA601, HA602, HA603, HA604, HA605 |
| Stroke-related procedures | (Percutaneous thrombectomy) M6630, M6631, M6632, M6633 |
| IHD/AMI-related diagnostic tests | (Coronary angiography) HA670, HA680, HA681, HA682 |
| IHD/AMI-related procedures | (Percutaneous Coronary Intervention, PCI) M6551, M6552, M6553, M6554, M6561, M6562, M6563, M6564, M6565, M6566, M6567, M6571, M6572<br>(Percutaneous thrombectomy) M6633, M6634<br>(Coronary Artery Bypass Graft, CABG) O1641, O1642, O1647, O1648, O1649, OA640, OA641, OA642, OA647, OA648, OA649 |
| Laboratory data | (Creatine kinase-MB, Troponin levels) B2640, C3941, C3942, CY277, CY278, CY279, D4040, D4023, D4021, D402 |

IHD: ischemic heart disease, AMI: acute myocardial infarction

Supplementary Table S2. Distribution of non-radiation risk factors among the total study population and by subtypes of circulatory diseases

| | Total population | IS | HS | IHD | AMI | VHD | HF |
|---|---|---|---|---|---|---|---|
| | Number (%) | Number (%) | Number (%) | Number (%) | Number (%) | Number (%) | Number (%) |
| Population at risk | 186 233 (100.0) | 186 030 (100.0) | 186 079 (100.0) | 184 522 (100.0) | 185 908 (100.0) | 185 966 (100.0) | 185 559 (100.0) |
| Cases | - | 1888 | 775 | 8562 | 1713 | 788 | 5892 |
| Income[a] | | | | | | | |
| Missing | 330 (0.2) | 2 (0.1) | 1 (0.1) | 7 (0.1) | 3 (0.2) | 2 (0.2) | 7 (0.1) |
| Low | 34 556 (18.6) | 379 (19.8) | 166 (21.1) | 1431 (15.9) | 333 (18.8) | 118 (14.1) | 1038 (17.3) |
| Medium | 66 423 (35.7) | 635 (33.2) | 268 (34.0) | 2656 (29.5) | 522 (29.5) | 241 (28.8) | 1861 (31.1) |
| High | 84 924 (45.6) | 895 (46.8) | 353 (44.8) | 4913 (54.5) | 914 (51.6) | 476 (56.9) | 3079 (51.4) |
| Smoking | | | | | | | |
| Missing | 17 304 (9.3) | 203 (10.6) | 113 (14.3) | 542 (6.0) | 160 (9.0) | 53 (6.3) | 413 (6.9) |
| Never | 75 796 (40.7) | 631 (33.0) | 247 (31.3) | 3057 (33.9) | 517 (29.2) | 392 (46.8) | 2148 (35.9) |
| Former | 24 015 (12.9) | 278 (14.5) | 109 (13.8) | 1491 (16.6) | 252 (14.2) | 136 (16.2) | 938 (15.7) |
| Current | 69 118 (37.1) | 799 (41.8) | 319 (40.5) | 3917 (43.5) | 843 (47.6) | 256 (30.6) | 2486 (41.5) |
| BMI | | | | | | | |
| Missing | 17 253 (9.3) | 203 (10.6) | 112 (14.2) | 539 (6.0) | 161 (9.1) | 53 (6.3) | 410 (6.9) |
| < 18.5 | 6 536 (3.5) | 27 (1.4) | 13 (1.6) | 163 (1.8) | 24 (1.4) | 27 (3.2) | 119 (2.0) |
| 18.5-24.9 | 107 236 (57.6) | 1009 (52.8) | 395 (50.1) | 4726 (52.5) | 854 (48.2) | 483 (57.7) | 3098 (51.8) |
| 25.0-29.9 | 47 781 (25.7) | 604 (31.6) | 231 (29.3) | 3176 (35.3) | 653 (36.9) | 246 (29.4) | 2023 (33.8) |
| ≥ 30.0 | 7427 (4.0) | 68 (3.6) | 37 (4.7) | 403 (4.5) | 80 (4.5) | 28 (3.3) | 335 (5.6) |
| Blood pressure[b] | | | | | | | |
| Missing | 17 261 (9.3) | 205 (10.7) | 112 (14.2) | 544 (6.0) | 160 (9.0) | 53 (6.3) | 414 (6.9) |
| Normal | 63 554 (34.1) | 327 (17.1) | 140 (17.8) | 1925 (21.4) | 340 (19.2) | 196 (23.4) | 1291 (21.6) |
| Pre-hypertension | 82 941 (44.5) | 789 (41.3) | 319 (40.5) | 4048 (44.9) | 774 (43.7) | 363 (43.4) | 2664 (44.5) |
| Hypertension | 22 477 (12.1) | 590 (30.9) | 217 (27.5) | 2490 (27.6) | 498 (28.1) | 225 (26.9) | 1616 (27.0) |
| Blood glucose (mg/L) | | | | | | | |
| Missing | 17 261 (9.3) | 204 (10.7) | 112 (14.2) | 544 (6.0) | 159 (9.0) | 53 (6.3) | 415 (6.9) |
| < 100 | 128 795 (69.2) | 1000 (52.3) | 447 (56.7) | 5482 (60.9) | 992 (56.0) | 570 (68.1) | 3624 (60.6) |
| 100~125 | 34 236 (18.4) | 475 (24.9) | 175 (22.2) | 2194 (24.4) | 422 (23.8) | 168 (20.1) | 1444 (24.1) |
| ≥ 126 | 5941 (3.2) | 232 (12.1) | 54 (6.9) | 787 (8.7) | 199 (11.2) | 46 (5.5) | 502 (8.4) |

[a]0(medical aid and beneficiary)–6th quantile of insurance amount, low; 7th–13th quantiles, medium; and 14th–20th quantiles, high.

[b]<120 mm Hg of systolic BP and <80 mm Hg for diastolic BP, normal; 120–140 mm Hg of systolic BP or 80–90 mm Hg for diastolic BP, pre-hypertension; and ≥140 mm Hg of systolic BP or ≥90 mm Hg for diastolic BP, hypertension

IS: ischemic stroke, HS: hemorrhagic stroke, IHD: ischemic heart disease, AMI: acute myocardial infarction, VHD: valvular heart disease, HF: heart failure, BMI: body mass index

Supplementary Table S3. Relative rate (RR)[a] and excess relative risk (ERR)[b] for combined stroke and heart disease outcomes by cumulative heart or brain absorbed dose (10-year lag)

| | Stroke | | Heart disease | |
|---|---|---|---|---|
| Cases | 2481 | | 12 836 | |
| Person-years | 2 398 240 | | 2 387 123 | |
| Cumulative dose (mGy) | RR | 95% CI | RR | 95% CI |
| 0 | 1 | | 1 | |
| ≤ 1 | 0.82 | 0.74–0.92 | 0.87 | 0.83–0.91 |
| >1–2.5 | 0.84 | 0.73–0.95 | 0.84 | 0.79–0.89 |
| >2.5–5.0 | 0.85 | 0.74–0.99 | 0.84 | 0.78–0.89 |
| >5.0–10.0 | 0.96 | 0.83–1.12 | 0.86 | 0.80–0.92 |
| >10.0–20.0 | 0.86 | 0.71–1.04 | 0.85 | 0.77–0.92 |
| >20.0–50.0 | 0.96 | 0.77–1.19 | 0.87 | 0.78–0.96 |
| >50.0 | 0.83 | 0.60–1.15 | 0.84 | 0.72–0.97 |
| | ERR | 95 % CI | ERR | 95 % CI |
| ERR per 10 mGy with 10-y lagged | -0.008 | -0.040–0.023 | -0.016 | -0.228–0.002 |

[a]Adjusted for sex, attained age, calendar year, and birth year

[b]Estimates based on linear model, adjusted for sex, attained age, calendar year, and birth year

Supplementary Table S4. Estimated dose coefficients in linear (per 10 mGy with 10-y lagged) and linear-quadratic ERR[a] models for circulatory disease subtypes

| | Linear | | Linear quadratic | | | | P-value |
|---|---|---|---|---|---|---|---|
| | | | Linear | | Quadratic | | |
| | Coeff. | 95% CI | Coeff. | 95% CI | Coeff. | 95% CI | |
| IS | -0.016 | -0.050–0.018 | -0.020 | -0.117–0.077 | 0.005 | -0.114–0.124 | > 0.50 |
| HS | 0.014 | -0.049–0.076 | 0.094 | -0.079–0.266 | -0.115 | -0.330–0.101 | 0.283 |
| IHD | -0.008 | -0.025–0.009 | -0.054 | -0.099– -0.010 | 0.065 | 0.007–0.123 | 0.028 |
| AMI | -0.013 | -0.049–0.023 | -0.056 | -0.152–0.041 | 0.059 | -0.066–0.184 | 0.344 |
| VHD | -0.019 | -0.075–0.037 | -0.043 | -0.196–0.109 | 0.033 | -0.162–0.228 | > 0.50 |
| HF | -0.020 | -0.039– -0.001 | -0.073 | -0.125– -0.021 | 0.071 | 0.004–0.137 | 0.035 |

[a]Adjusted for sex, attained age, calendar year, and birth year

Supplementary Table S5. Risks for circulatory disease subtypes by working duration

| | IS | | HS | | IHD | | AMI | | VHD | | HF | |
|---|---|---|---|---|---|---|---|---|---|---|---|---|
| | SIR | 95% CI | SIR | 95% CI | SIR | 95% CI | SIR | 95% CI | SIR | 95% CI | SIR | 95% CI |
| Full cohort | 0.73 | 0.70–0.77 | 0.70 | 0.65–0.75 | 0.96 | 0.93–0.98 | 0.90 | 0.85–0.95 | 0.98 | 0.91–1.06 | 0.89 | 0.86–0.91 |
| Duration < 1 year (mean dose: heart 0.87, brain 0.85 mGy) | 0.94 | 0.87–1.01 | 0.93 | 0.83–1.04 | 1.07 | 1.03–1.11 | 1.04 | 0.96–1.13 | 1.10 | 0.97–1.25 | 1.00 | 0.96–1.05 |
| Duration ≥ 1 year (mean dose: heart 5.81, brain 5.53 mGy) | 0.63 | 0.59–0.67 | 0.58 | 0.53–0.65 | 0.90 | 0.87–0.92 | 0.82 | 0.77–0.88 | 0.92 | 0.84–1.02 | 0.83 | 0.80–0.86 |
| | ERR[a] per 10 mGy | 95% CI | ERR[a] per 10 mGy | 95% CI | ERR[a] per 10 mGy | 95% CI | ERR[a] per 10 mGy | 95% CI | ERR[a] per 10 mGy | 95% CI | ERR[a] per 10 mGy | 95% CI |
| Full cohort | -0.02 | -0.05–0.02 | 0.01 | -0.05–0.08 | -0.01 | -0.03–0.01 | -0.01 | -0.05–0.02 | -0.02 | -0.08–0.04 | -0.02 | -0.04– -0.001 |
| Duration < 1 year (mean dose: heart 0.87, brain 0.85 mGy) | 0.34 | -0.13–0.80 | 0.40 | -0.29–1.08 | -0.06 | -0.11– -0.02 | -0.06 | -0.22–0.10 | -0.05 | -0.54–0.44 | -0.06 | -1.16– -0.10 |
| Duration ≥ 1 year (mean dose: heart 5.81, brain 5.53 mGy) | 0.02 | -0.02–0.07 | 0.07 | -0.02–0.15 | 0.01 | -0.01, 0.03 | 0.002 | -0.04–0.04 | -0.01 | -0.07–0.05 | -0.01 | -0.03– 0.02 |

[a]Estimates based on linear model, adjusted for sex, attained age, calendar year, and birth year

IS: ischemic stroke, HS: hemorrhagic stroke, IHD: ischemic heart disease, AMI: acute myocardial infarction, VHD: valvular heart disease, HF: heart failure, ERR: excess relative risk, SIR: standardized incidence ratio, CI: confidence interval
